# Potential impact of climate change on emerging vector-borne and other infections in the UK

**DOI:** 10.1186/s12940-017-0326-1

**Published:** 2017-12-05

**Authors:** Matthew Baylis

**Affiliations:** 1 Liverpool University Climate and Infectious Diseases of Animals group, Institute of Infection and Global Health, University of Liverpool, Liverpool, UK; 2 Health Protection Research Unit in Emerging and Zoonotic Infections, University of Liverpool, Liverpool, UK

**Keywords:** Climate, Climate change, Disease, Vector, Mosquito, Tick, Sandfly, West nile, Lyme, malaria, Zika

## Abstract

Climate is one of several causes of disease emergence. Although half or more of infectious diseases are affected by climate it appears to be a relatively infrequent cause of human disease emergence. Climate mostly affects diseases caused by pathogens that spend part of their lifecycle outside of the host, exposed to the environment. The most important routes of transmission of climate sensitive diseases are by arthropod (insect and tick) vectors, in water and in food. Given the sensitivity of many diseases to climate, it is very likely that at least some will respond to future climate change. In the case of vector-borne diseases this response will include spread to new areas. Several vector-borne diseases have emerged in Europe in recent years; these include vivax malaria, West Nile fever, dengue fever, Chikungunya fever, leishmaniasis, Lyme disease and tick-borne encephalitis. The vectors of these diseases are mosquitoes, sand flies and ticks. The UK has endemic mosquito species capable of transmitting malaria and probably other pathogens, and ticks that transmit Lyme disease. The UK is also threatened by invasive mosquito species known to be able to transmit West Nile, dengue, chikungunya and Zika, and sand flies that spread leishmaniasis. Warmer temperatures in the future will increase the suitability of the UK’s climate for these invasive species, and increase the risk that they may spread disease. While much attention is on invasive species, it is important to recognize the threat presented by native species too. Proposed actions to reduce the future impact of emerging vector-borne diseases in the UK include insect control activity at points of entry of vehicles and certain goods, wider surveillance for mosquitoes and sand flies, research into the threat posed by native species, increased awareness of the medical profession of the threat posed by specific diseases, regular risk assessments, and increased preparedness for the occurrence of a disease emergency.

**Electronic supplementary material:**

The online version of this article (10.1186/s12940-017-0326-1) contains supplementary material, which is available to authorized users.

## Background

There is widespread scientific agreement that anthropogenic activities are contributing to the warming of the world’s climate at an unprecedented rate, with concomitant changes in precipitation, flooding, winds and the frequency of extreme events. Many studies have demonstrated links between infectious diseases and climate, and it is highly likely that changes in climate will impact on at least some of them. This review summarises the state of scientific evidence regarding current and future impacts of anthropogenic climate change on the emergence of infectious diseases with potential to impact significantly on the UK human population. Infections are defined as ‘emerging’ if they “have newly appeared in a population or have existed previously but are rapidly increasing in incidence or geographic range” [1]. Initially the review considers a broad range of possible emerging infections but then narrows the focus to vector-borne diseases.

### Emerging diseases

Human diseases are affected by climate when the pathogen spends a significant period of time outside of the human host, subject to environmental influence. The most climate-sensitive diseases are, therefore, those spread by arthropod vectors, in food or water, in aerosol, in fomites, and those with free-living stages.

By contrast, most diseases transmitted directly between humans (for example, human childhood viruses, sexually transmitted diseases, tuberculosis) have few or no reported associations with climate. Exceptions are the human respiratory viruses, such as those that cause colds and seasonal flu, which spread by aerosol between individuals in close contact but are nevertheless sensitive to the effects of ambient humidity and possibly temperature, and have strong seasonal cycles.

Systematic reviews of disease-climate associations support these assertions. Scrutiny of research papers on 157 high-impact human and animal pathogens of Europe found that 66% have at least one climate variable that affects disease occurrence. Transmission routes of climate sensitive diseases, in decreasing order of importance, were vectors, food/water, environment, fomite and aerosol [2]. Similarly, a weighted risk analysis following systematic review identified 26 (49%) out of 53 reportable infectious diseases of humans in Europe to be directly or indirectly linked to climate [3].

Climate affects three major aspects of disease occurrence: where, when and how much. In some cases, mostly macroparasite infections caused by helminths, climate can also affect the severity of clinical disease in infected individuals.

Vector-borne diseases usually have geographically-restricted distributions because of the effects of climate of the insects/ticks. Such diseases are particularly likely, therefore, to spread to new areas with climate change. Some water-borne diseases may also spread in response to climate change, while the response of other climate-sensitive diseases may be change to their seasonal cycles or the frequency or scale of disease outbreaks in endemic regions.

Extreme weather events are an important factor in the spread of water-borne diseases such as cholera, leptospirosis and gastrointestinal infections. A systematic review [4] found that heavy rainfall and flooding frequently precede water-borne disease outbreaks. At the other extreme, drought can lead to the concentration of water-borne pathogens in rivers and water bodies. Temperature is also important: higher temperatures lead to faster growth rates of disease-causing pathogens, and increased use of bathing waters by the public [5].

Climate change is just one of several forces (“drivers”) that may lead to the emergence of infectious diseases. Other drivers are changes to the environment through deforestation and urbanization; developments in agriculture and food production; changes in how people live, behave, eat, travel and trade; changes in medicine, public health and the use of antimicrobials; and the occurrence of “shocks” such as war, migration and famine [6]. An analysis of more than 300 human disease outbreaks concluded that climate and weather were infrequent causes (3%) [6]. Some other drivers, however, such as changes to land use and agriculture (11%), are themselves affected by climate change. Therefore, climate change’s indirect effects on disease emergence (via effects on other drivers) may also be important.

There are, therefore, strong arguments to expect climate change to impact on human disease outbreaks, especially those transmitted by vectors or in food/water, but in practice there are relatively few examples for which there is documented evidence. Recently, however, Europe has seen an upsurge in vector-borne diseases, as well as the spread of vectors, and the possible role of climate change needs consideration.

### Recent impact of climate change on vector-borne disease emergence in Europe

The main vectors threatening or affecting the health of people in Europe are mosquitoes, sandflies and ticks.

Malaria, caused by *Plasmodium vivax* and spread by the bites of *Anopheles* mosquitoes, used to be endemic the UK but there has been no transmission via native mosquitoes for over 60 years [7]. The disappearance of malaria is attributed largely to changes to the environment (drainage of marshes) and farming [7]. Similar changes have occurred in mainland Europe and no areas are now endemic for the disease. In 2009–2012, however, outbreaks of *P. vivax* malaria occurred in Greece, after nearly 40 years of only sporadic cases. This is not linked to climate change: the origins are likely to be migrant workers introducing *P. vivax* into an agricultural region where vectors were present [8]. Nevertheless, environmental risk modelling showed that the regions of Greece affected by malaria in 2009–2012 could be successfully predicted using digital elevation (i.e. altitude) and satellite-derived Land Surface Temperature variables, indicating an important role of temperature in determining where transmission is possible, following an introduction [9].

West Nile fever, caused by West Nile virus (WNV) and spread by *Culex* mosquitoes, is endemic in a number of southern and eastern European countries. The incidence of human cases is increasing [10]. WNV vectors are very sensitive to changes in temperature [11], and increasing temperature is considered to be a driver of its emergence in Europe [12]. In the UK there have been only unconfirmed reports of the transmission of WNV among birds. However, a known WNV vector, *Culex modestus,* was recently rediscovered in southern England, after no reports in the UK since the 1940s [13]. This re-establishment has not been documented to be linked to climate change, but it is interesting to note that the 1940s were the warmest recorded period in England (Central England time series) until the present [14].

Indigenous UK mosquito species may present an underestimated risk for the transmission of arboviruses. For a mosquito to act as a vector, it needs to live longer than the time required for a pathogen to develop within it, and native species may be more able to survive (and hence live longer) at the UK’s relatively cool temperatures than exotic species. There is a good example from the field of animal disease: bluetongue, a midge-borne viral disease of ruminants, is transmitted in Mediterranean countries by an invasive, African midge species. It was not foreseen that indigenous midges could transmit bluetongue in climates as cool as southern Scandinavia. In 2006–2009, tens of thousands of farms across northern Europe, including some in the UK, were affected by this disease [15]. It is noteworthy, therefore, that a UK native mosquito, *Ochleratatus detritus,* which feeds readily on humans, is a good vector of flaviviruses, the group that includes WNV [16].

Invasive mosquitoes also present a risk of the introduction of new diseases. There have been several outbreaks of Chikungunya fever and dengue in north Italy, southern France and Croatia since 2007 [17–21]. The vector is the Asian tiger mosquito, *Aedes albopictus*, an invasive species that has spread from Asia to North America and Europe, where it is now found in most southern European countries but also Belgium and The Netherlands. The major dengue vector, *Ae. aegypti*, disappeared from Europe in the mid-twentieth century but has now returned, being found in Madeira (an autonomous region of Portugal) in 2004–05 [22], and in the far south-east (Georgia, Abkhazia, Sochi region of Russia) [23]. Despite vector control efforts in Madeira, the mosquito has thrived; and in 2012 there were more than 2000 dengue cases, the first sustained transmission of dengue in Europe since the 1920s [24].

The emergence of dengue and Chikungunya in Europe is associated with the establishment of *Ae. albopictus* and *Ae. aegypti*, together with mutation of Chikungunya virus that facilitated infection of *Ae. albopictus*. The mosquitoes became infected after feeding on returning travellers carrying infections obtained overseas. The emergence is, therefore, not directly associated with climate change although climate has undoubtedly played an important role in determining the geographic area where transmission is possible, and climate change may have extended that range [25].

There is a significant risk that Zika virus, which is believed to have infected over a million people in South America in 2015–2016, will emerge in Europe in 2016 or 2017. Both *Ae. aegypti* and *Ae. albopictus* are competent vectors [26] and the greatest risk is where these vectors are present. Most studies find the vector competence of *Ae. aegypti* for Zika virus to be higher than that of *Ae. albopictus* and this, combined with the greater anthropophily and biting rates of the former [27], suggests that the risk of Zika transmission in Europe is highest where *Ae. aegypti* is present (i.e. Madeira, Georgia), and lower where only *Ae. albopictus* is present. There may be other mosquitoes in UK/Europe capable of transmitting Zika virus that we are currently unaware of. Zika virus can also be spread sexually [28], and there is a small risk of transmission outside of areas with competent vectors [29].

Sandflies are vectors of cutaneous and visceral leishmaniasis in people and dogs in southern Europe. There is a low incidence in people (~700 cases per year in southern Europe) but a high incidence in dogs (about 5000 cases per year in France alone) [30]. Sandflies have not been detected in the UK but have been found in central and northern France and southern Germany [31]. The presence of known vectors in northern Europe, and the passport-controlled movement of potentially infected dogs into the UK [32], raises the possibility of the eventual introduction of leishmaniasis.

Two tick-borne diseases have emerged in Europe in recent decades, tick-borne encephalitis (TBE) and Lyme disease, both transmitted by the sheep tick, *Ixodes ricinus.* The emergence of TBE is a major concern [33]. There are thousands of cases of human disease each year, the incidence is increasing, and it is spreading to new regions. Possible causes of this emergence are socioeconomic, environmental and climatic changes, as well as increased awareness [33, 34]. The role of climate change on TBE emergence in the Baltics is contested [35] and other drivers of disease emergence, such as change in human behavior, are believed to be important factors in this region [36].

While TBE occurs as far north as Scandinavia (indicating that the UK climate should not be a barrier to disease), and the *I. ricinus* vector is found throughout the British Isles, TBE virus has not been reported in the UK. This may be because *I. ricinus* transmits the related Louping ill virus in the UK, which causes disease in sheep and only rarely affects people.

A bacterial spirochaete disease, called Lyme borreliosis and also spread by *I. ricinus*, is emerging in the UK. Lyme disease is the most common vector-borne disease in temperate climates, with an estimated 85,000 cases per year in Europe; over 2000 cases occur per year in the UK alone. Lyme disease is increasing in incidence, possibly because of climate-change influences on the tick vector. Increased *I. ricinus* density and its spread to higher latitude in Sweden have been linked to milder winters due to climate change [37].

Tick-borne disease threats also include Crimean Congo Haemorrhagic Fever (CCHF). This viral disease is spread largely by *Hyalomma* ticks, is endemic in parts of Eastern and south-eastern Europe; and it appears to be emerging [38]. *Hyalomma* ticks are not endemic to the UK although they may arrive on migrant birds [39]; and CCHF virus may arrive in infected travellers [40].

Most consideration has been given here to vector-borne diseases but it is important to note that many non-vector-borne diseases have emerged in recent years. Of 38 human pathogens listed by Public Health England as having emerged (globally) since 1980, only 4 are vector-borne. The emergence of 25 of these 38 pathogens is included in a study of drivers of emergence [6] and none were attributed to climate. In other words, the majority of emerging diseases are not vector-borne, and their emergence is not linked to climate change.

### Future impact of climate change on vector-borne disease emergence in Europe

The World Health Organization has attempted to quantify the additional amount of human disease that might arise as a consequence of climate change [41, 42]. Inevitably, there is significant uncertainty. In a recent assessment for the years 2030 and 2050, WHO found increases of 20–86,000 deaths globally from diarrhoeal disease of children attributable to climate change, under a range of socioeconomic growth scenarios [42]. However, the number in Western Europe was very small (1 or 2). Equally, climate change led to no projected deaths in Western Europe from *P. falciparum* malaria or dengue.

Several other workers have developed models of climate change’s future impacts on malaria. The most extensive examined the projections of five malaria impact models, each driven by five global climate models and four emission scenarios [43]. The probability of sustained malaria transmission occurring in the UK in the current century was found to be small, even under the most extreme emission scenario.

It is important to note, however, that all five malaria models were developed and parameterized for malaria caused by *P. falciparum*, while it was malaria from *P. vivax* that was previously endemic in northern Europe, including the UK, and which has recently reoccurred in Europe. *Plasmodium vivax* transmission by European vectors requires lower temperature thresholds than *P. falciparum* and, therefore, all five malaria models and WHO’s assessments underestimate the risk of malaria in Europe. By contrast, under a medium-high scenario of climate change, a model parameterized for *P. vivax* predicts that the southern half of Great Britain will be climatically-suitable for *P. vivax* malaria transmission by 2030 for 2 months of the year, and parts of the southeast of England for 4 months per year [44]; while by 2080 even southern Scotland will be climatically-suitable for 2 months of the year [45].

The risk of malaria transmission is, however, determined by many factors other than climate; and while climate change may increase the suitability of the UK for transmission, other factors (such as drainage of marshes and changes in land use) make the probability of it occurring small.

The main threat to the UK from dengue, chikungunya and Zika viruses is tied to the risk of invasion by *Ae. aegypti* and *Ae. albopictus*. The suitability of the current European climate for both vectors and dengue transmission has been modelled [46]. Much of Europe, including many parts of the UK, were found to be suitable for *Ae. albopictus*; however, few areas of Europe, and none in the UK, were suitable for *Ae. aegypti*. Other workers report similar findings [25]. Three models indicate that large parts of the UK are already suitable for *Ae. albopictus* and predict increased climatic suitability in future. Importantly, in autumn 2016 *Ae. albopictus* eggs were collected for the first time in the UK (southern England) [47].

Arbovirus transmission requires that environmental conditions are suitable for both virus and vector, and even where vectors are present, it may be too cold for viral transmission. Studies of the climatic needs of chikungunya virus (based on conditions at past outbreaks) indicate a threshold minimum temperature of 20–22 **°**C. Combining this threshold with the climatic needs of the vector, the UK is found to be climatically unsuitable for chikungunya transmission at the present time [48] although some parts of England may alter to become ‘rather unsuitable’ (the second lowest of five risk classes) later in the century. No parts of the UK become suitable for dengue virus development (in *Aedes aegypti*) by 2100 [49]. There is not consensus, however, Other models of vectorial capacity find that under the most extreme emission scenarios there is a risk of transmission of dengue by *Ae. aegypti* (and to a lesser extent, *Ae. albopictus*) during the UK summer by 2100 [50, 51].

While there is consensus that climate affects the transmission of tick-borne Lyme borreliosis, and some agreement that the northward spread of the vector in Sweden is attributable to recent warming, there are no published models projecting its future incidence in Europe under scenarios of climate change. Models for other temperate regions predict that climate change will allow northern expansion of the range of the tick vectors [52]. Lyme and its vectors are already found throughout the UK and, therefore, increasing climate suitability may be limited to increase in altitude [53] and change in incidence. Non-climate drivers, however, play a dominant role in the epidemiology of Lyme disease [54] and future trends in agriculture, land use, wild animal populations and tourism will play a large role in determining future patterns of the disease [55].

### Potential for impacts to be avoided by adaptation measures

Adaptation to the risk of emerging infections requires three actions: (i) improved surveillance of disease and, where appropriate, vectors, so that sporadic cases and outbreaks, or the heightened risk of these, can be detected early; (ii) risk assessments of the likelihood of cases or outbreaks of an emerging disease, regularly updated as new data become available, including forecasts based on vector, disease, climate and sociodemographic and travel/transport data; and (iii) increased preparedness for outbreaks.

The northward spread of *Ae. albopictus* indicates the need for active surveillance for invasive mosquitoes. There is strong evidence that some of the spread of *Ae. albopictus* in continental Europe is associated with transport in vehicles as, for example, it has been trapped in motorway service areas. The extensive movement of cars and goods vehicles into the UK from continental Europe should be considered a likely route of entry of this mosquito. Insecticidal treatment of vehicles entering the UK could help to reduce this risk. ‘Disinsection’, as described by the International Health Regulations of the WHO, is already used to kill insects in airplanes arriving in certain countries. *Aedes albopictus* has also entered many countries in used vehicle tyres or in ‘lucky bamboo’, a houseplant imported from China. Mosquito surveillance at ports of entry of such products is recommended.

A known vector of West Nile virus, *Culex modestus*, has re-established in the UK. Surveillance within affected regions can help identify areas of particular risk of virus transmission; and surveillance outside such areas can be used to monitor its further spread.

Adaptation measures to Lyme disease include increasing awareness of the public, in terms of recognizing symptoms and taking tick-avoidance measures; and improving national-level surveillance. For surveillance, it has been recommended to base the clinical definition of Lyme disease on the skin rash that occurs in about 90% of cases, rather than on the rare Lyme neuroborreliosis that needs laboratory confirmation [3]. Forecasts of the risk of exposure to tick vectors or Lyme, based on geographic and climatic information, will help the public to take suitable precautions.

The risk of emerging disease outbreaks in the UK, particularly vector-borne, food-borne and water-borne diseases, is likely to continue to rise as our climate changes. New data will frequently become available, such as updated maps of the occurrence of potentially invasive vectors in continental Europe. Risk assessments should, therefore, be regularly updated.

Preparedness for outbreaks of vector-borne diseases requires (i) knowledge of the vectors, (ii) identification of areas of high risk, (iii) in such areas, raised awareness of the public and health professionals and (iv) development of policy and approval of legislation to permit vector control programmes. A contingency plan should be developed for the event that *Ae. albopictus* is detected in the UK.

## Conclusions

Given the sensitivity of many diseases to climate, it is very likely that at least some will respond to future climate change. Many water-borne and food-borne diseases are climate sensitive but there is a relative paucity of published studies on the impacts of climate change upon them. The impact of climate change on infectious respiratory diseases requires further study, as such diseases can spread rapidly and cause significant health impact; detailed studies of the impacts of climate change on them, however, has received little attention [56].

This review has focused extensively on vector-borne diseases because they are particularly climate-sensitive. Several vector-borne diseases have recently emerged in Europe, and some threaten the UK. The greatest risk is probably presented by West Nile virus, as is widespread in southern and Eastern Europe and the UK harbours at least one competent vector species. There is a threat of invasion by the Asian Tiger mosquito, *Ae. albopictus*; if the vector enters and survives in the UK, however, it may be too cold for sustained transmission of dengue, chikungunya or Zika viruses. The UK is, and long has been, climatically suitable for the transmission of malaria caused by *P. vivax*. Climate change will increase the climatic suitability even further, but this is unlikely to lead to sustained malaria transmission if land use and agricultural practices remain as they are today. However, the recreation of extensive wetlands and salt marshes, combined with warmer temperatures, may create a situation where local vivax malaria transmission is possible.

The emergence of Lyme disease in the UK is likely to continue in the future. Climate change may contribute to this emergence, but other drivers will probably play a larger role. The risk presented by TBE, which is emerging in many parts of Europe, remains difficult to gauge.

Key research gaps are: (i) assessment of the impact of climate change on diseases that are not vector-borne, including respiratory infections; (ii) evaluation of the vector potential of UK native species; (iii) development of climate driven models for the risk of West Nile and Lyme in the UK; (iv) evaluation of the threat posed by TBE to the UK and (v) incorporation of environmental, agricultural and sociodemographic variables into models of the risk of vivax malaria in the UK.

## Additional file

Additional file 1: Open peer review. (PDF 357 kb)
