# Supplementary material for: Potential impact of climate change on emerging vector-borne and other infections in the UK
Source: Environ Health. 2017 Dec 5;16(Suppl 1):112. doi: 10.1186/s12940-017-0326-1 (PMC5773876; doi:10.1186/s12940-017-0326-1)
Supplement: Additional file 1: — Open peer review. (PDF 357 kb) [file 12940_2017_326_MOESM1_ESM.pdf]

Reviewer reports

Title: Potential impact of climate change on emerging vector-borne and other infections in the UK

Reviewer 1: Jan Semenza
Competing interests declaration: I declare that I have no competing interests.

Potential impact of climate change on emerging infections in the UK

Matthew Baylis

Liverpool University Climate and Infectious Diseases of Animals group, Institute of Infection and Global Health, University of Liverpool; & Health Protection Research Unit in Emerging and Zoonotic Infections, University of Liverpool.

matthew.baylis@liv.ac.uk

Abstract
Climate is one of several causes of disease emergence. Although half or more of infectious diseases are affected by climate it appears to be a relatively infrequent cause of human disease emergence. Climate mostly affects diseases caused by pathogens that spend part of their lifecycle outside of the host, exposed to the environment. The most important routes of transmission of climate sensitive diseases are by arthropod (insect and tick) vectors, in water and in food. Given the sensitivity of many diseases to climate, it is very likely that at least some will respond to future climate change. In the case of vector-borne diseases this response will include spread to new areas. Several vector-borne diseases have emerged in Europe in recent years; these include vivax malaria, West Nile fever, dengue fever, Chikungunya fever, leishmaniasis, Lyme disease and tick-borne encephalitis. The vectors of these diseases are mosquitoes, sand flies and ticks. The UK has endemic mosquito species capable of transmitting malaria and probably other pathogens, and ticks that transmit Lyme disease. The UK is also threatened by invasive mosquito species known to be able to transmit West Nile, dengue, chikungunya and Zika, and sand flies that spread leishmaniasis. Warmer temperatures in the future will increase the suitability of the UK's climate for these invasive species, and increase the risk that they may spread disease. While much attention is on invasive species, it is important to recognize the threat presented by native species too. Proposed actions to reduce the future impact of emerging vector-borne diseases in the UK include insect control activity at points of entry of vehicles and certain goods, wider surveillance for mosquitoes and sand flies, research into the threat posed by native species, increased awareness of the medical profession of the threat posed by specific diseases, regular risk assessments, and increased preparedness for the occurrence of a disease emergency.

Keywords
Climate; Climate change; disease; vector; mosquito; tick; sandfly, West Nile, Lyme, malaria, Zika

Introduction

There is widespread scientific agreement that anthropogenic activities are contributing to the warming of the world's climate at an unprecedented rate, with concomitant changes in precipitation, flooding, winds and the frequency of extreme events. Many studies have demonstrated links between infectious diseases and climate, and it is highly likely that changes in climate will impact on at least some of them. This review summarises the state of scientific evidence regarding current and future impacts of anthropogenic climate change on the emergence of infectious diseases with potential to impact significantly on the UK human population. Infections are defined as 'emerging' if they "have newly appeared in a population or have existed previously but are rapidly increasing in incidence or geographic range" [1]. Initially the review considers a broad range of possible emerging infections but then narrows the focus to vector-borne diseases.

Emerging diseases

Human diseases are affected by climate when the pathogen spends a significant period of time outside of the human host, subject to environmental influence. The most climate-sensitive diseases are, therefore, those spread by arthropod vectors, in food or water, in aerosol, in fomites, and those with free-living stages.

By contrast, most diseases transmitted directly between humans (for example, human childhood viruses, sexually transmitted diseases, tuberculosis) have few or no reported associations with climate. Exceptions are the human respiratory viruses, such as those that cause colds and seasonal flu, which spread by aerosol between individuals in close contact but are nevertheless sensitive to the effects of ambient humidity and possibly temperature, and have strong seasonal cycles.

Systematic reviews of disease-climate associations support these assertions. Scrutiny of research papers on 157 high-impact human and animal pathogens of Europe found that 66% have at least one climate variable that affects disease occurrence. Transmission routes of climate sensitive diseases, in decreasing order of importance, were vectors, food/water, environment, fomite and aerosol [2]. Similarly, a weighted risk analysis following systematic review identified 26 (49%) out of 53 reportable infectious diseases of humans in Europe to be directly or indirectly linked to climate [3].

Climate affects three major aspects of disease occurrence: where, when and how much. In some cases, mostly macroparasite infections caused by helminths, climate can also affect the severity of clinical disease in infected individuals.

Vector-borne diseases usually have geographically-restricted distributions because of the effects of climate of the insects/ticks. Such diseases are particularly likely, therefore, to spread to new areas with climate change. Some water-borne diseases may also spread in response to climate change, while the response of other climate-sensitive diseases may be change to their seasonal cycles or the frequency or scale of disease outbreaks in endemic regions.

Extreme weather events are an important factor in the spread of water-borne diseases such as cholera, leptospirosis and gastrointestinal infections. A systematic review [4] found that heavy rainfall and flooding frequently precede water-borne disease outbreaks. At the other extreme, drought can lead to the concentration of water-borne pathogens in rivers and water bodies. Temperature is also important: higher temperatures lead to faster growth rates of disease-causing pathogens, and increased use of bathing waters by the public [5].

Climate change is just one of several forces ("drivers") that may lead to the emergence of infectious diseases. Other drivers are changes to the environment through deforestation and urbanization; developments in agriculture and food production; changes in how people live, behave, eat, travel and trade; changes in medicine, public health and the use of antimicrobials; and the occurrence of "shocks" such as war, migration and famine [6]. An analysis of more than 300 human disease outbreaks concluded that climate and weather were infrequent causes (3%). Some other drivers, however, such as changes to land use and agriculture (11%), are themselves affected by climate change. Therefore, climate change's indirect effects on disease emergence (via effects on other drivers) may also be important.

Comment [JS1]: Ref?

There are, therefore, strong arguments to expect climate change to impact on human disease outbreaks, especially those transmitted by vectors or in food/water, but in practice there are relatively few examples for which the evidence has been documented. Recently, however, Europe has seen an upsurge in vector-borne diseases, as well as the spread of vectors, and the possible role of climate change needs consideration.

Recent impact of climate change on vector-borne disease emergence in Europe

The main vectors threatening or affecting the health of people in Europe are mosquitoes, sandflies and ticks.

Malaria, caused by *Plasmodium vivax* and spread by the bites of *Anopheles* mosquitoes, used to be endemic the UK but there has been no transmission via native mosquitoes for over 60 years [7]. The disappearance of malaria is attributed largely to changes to the environment (drainage of marshes) and farming (Kuhn et al., 2003). Similar changes have occurred in mainland Europe and no areas are now endemic for the disease. In 2011, however, an outbreak of *P. vivax* malaria occurred in Greece, after nearly 40 years of only sporadic cases-. This is not linked to climate

Comment [JS2]: Ref.

change: the origin is likely to be migrant workers introducing *P. vivax* into an agricultural region where vectors were present [8].

West Nile fever, caused by West Nile virus (WNV) and spread by *Culex* mosquitoes, is endemic in a number of southern and eastern European countries. The incidence of human cases is increasing [9]. WNV vectors are very sensitive to changes in temperature [10], and increasing temperature is considered to be a driver of its emergence in Europe [11]. In the UK there have been only unconfirmed reports of the transmission of WNV among birds. However, a known WNV vector, *Culex modestus,* was recently rediscovered in southern England, after no reports in the UK since the 1940s [12]. This re-establishment has not been documented to be linked to climate change, but it is interesting to note that the 1940s were the warmest recorded period in England (Central England time series) until the present [13].

Indigenous UK mosquito species may present an underestimated risk for the transmission of arboviruses. For a mosquito to act as a vector, it needs to live longer than the time required for a pathogen to develop within it, and native species may be more able to survive (and hence live longer) at the UK's relatively cool temperatures than exotic species. There is a good example from the field of animal disease: bluetongue, a midge-borne viral disease of ruminants, is transmitted in Mediterranean countries by an invasive, African midge species. It was not foreseen that indigenous midges could transmit bluetongue in climates as cool as southern Scandinavia. In 2006-2009, tens of thousands of farms across northern Europe, including some in the UK, were affected by this disease [14]. It is noteworthy, therefore, that a UK native mosquito, *Ochleratatus detritus,* which feeds readily on humans, is a good vector of flaviviruses, the group that includes WNV [15].

Invasive mosquitoes also present a risk of the introduction of new diseases. There have been several outbreaks of Chikungunya fever and dengue in north Italy, southern France and Croatia since 2007 [16-20]. The vector is the Asian tiger mosquito, *Aedes albopictus*, an invasive species that has spread from Asia to North America and Europe, where it is now found in most southern European countries but also Belgium and The Netherlands. The major dengue vector, *Ae. aegypti*, disappeared from Europe in the mid-20th century but has now returned, being found in Madeira (an autonomous region of Portugal) in 2004-05 [21]. Despite vector control efforts, the mosquito has thrived; and in 2012 there were more than 2,000 dengue cases, the first sustained transmission of dengue in Europe since the 1920s [22].

The emergence of dengue and Chikungunya in Europe is associated with the establishment of *Ae. albopictus* and *Ae. aegypti*, together with mutation of Chikungunya virus that facilitated infection of *Ae. albopictus*. The mosquitoes became infected after feeding on returning travellers carrying infections obtained overseas. The ~~emergence~~ introduction is, therefore, not associated with climate change.

There is a significant risk that Zika virus, which is believed to have infected over a million people in South America in 2015-2016, will emerge in Europe in 2016. Both *Ae. aegypti* and *Ae. albopictus* are competent vectors [23] and the greatest risk is where these vectors are present. However, Zika virus can also be spread sexually

Comment [JS3]: The introdcution is obviously necessary but not necessarely sufficient for onward transmission. We showed that temperture was one of the environmental determinants. Please see: Sudre B, Rossi M, van Bortel W., Danis K, Baka A, Vakalis N, Semenza JC. Mapping environmental suitability of malaria transmission in Greece. Emerg Infect Dis. 2013; 19(5):784-6

Comment [JS4]: There is a difference between a lack of association and lack of evidence of an association. Are there any studies that have attempted to establish an association?

Comment [JS5]: but the onward transmission might be. Has anyone looked at that?

[24], and there is a small risk of transmission outside of areas with competent vectors.

Sandflies are vectors of cutaneous and visceral leishmaniasis in people and dogs in southern Europe. There is a low incidence in people (~700 cases per year in southern Europe) but a high incidence in dogs (about 5,000 cases per year in France alone) [25]. Sandflies have not been detected in the UK but have been found in central and northern France and southern Germany [26]. The presence of known vectors in northern Europe, and the passport-controlled movement of potentially infected dogs into the UK [27], raises the possibility of the eventual introduction of leishmaniasis.

Two tick-borne diseases have emerged in Europe in recent decades, tick-borne encephalitis (TBE) and Lyme disease, both transmitted by the sheep tick, *Ixodes ricinus.* The emergence of TBE is a major concern [28]. There are thousands of cases of human disease each year, the incidence is increasing, and it is spreading to new regions. Possible causes of this emergence are socioeconomic, environmental and climatic changes, as well as increased awareness (Stefanoff, 2012 #3038; Lukan, 2010 #3026}. The role of climate change is contested [29] and other drivers of disease emergence, such as change in human behavior, believed to be important [30].

While TBE occurs as far north as Scandinavia (indicating that the UK climate should not be a barrier to disease), and the *I. ricinus* vector is found throughout the British Isles, TBE virus has not been reported in the UK. This may be because *I. ricinus* transmits the related Louping ill virus in the UK, which causes disease in sheep and only rarely affects people.

A bacterial spirochaete disease, called Lyme borreliosis and also spread by *I. ricinus*, is emerging in the UK. Lyme disease is the most common vector-borne disease in temperate climates, with an estimated 85,000 cases per year in Europe; over 2,000 cases occur per year in the UK alone. Lyme disease is increasing in incidence, possibly because of climate-change influences on the tick vector. Increased *I. ricinus* density and its spread to higher latitude in Sweden have been linked to milder winters due to climate change [31].

Tick-borne disease threats also include Crimean Congo Haemorrhagic Fever (CCHF). This viral disease is spread largely by *Hyalomma* ticks, is endemic in parts of Eastern and south-eastern Europe; and it appears to be emerging [32]. *Hyalomma* ticks are not endemic to the UK although they may arrive on migrant birds [33]; and CCHF virus may arrive in infected travellers [34].

Most consideration has been given here to vector-borne diseases but it is important to note that many non-vector-borne diseases have emerged in recent years. Of 38 human pathogens listed by Public Health England as having emerged (globally) since 1980, only 4 are vector-borne. The emergence of 25 of these 38 pathogens is included in a study of drivers of emergence [6] and none were attributed to climate. In other words, the majority of emerging diseases are not vector-borne, and their emergence is not linked to climate change.

Comment [JS6]: Refs

Comment [JS7]: on TBE emergence in the Baltic

Comment [JS8]: factors in the Baltic states.

Comment [JS9]: Ref 6 is an international assessment. There are more recent European studies that are more relevant.

Future impact of climate change on vector-borne disease emergence in Europe

The World Health Organization has attempted to quantify the additional amount of human disease that might arise as a consequence of climate change [35, 36]. Inevitably, there is significant uncertainty. In a recent assessment for the years 2030 and 2050, WHO found increases of 20 - 86,000 deaths globally from diarrhoeal disease of children attributable to climate change, under a range of socioeconomic growth scenarios [36]. However, the number in Western Europe was very small (1 or 2). Equally, climate change led to no projected deaths in Western Europe from *P. falciparum* malaria or dengue.

Several other workers have developed models of climate change's future impacts on malaria. The most extensive examined the projections of five malaria impact models, each driven by five global climate models and four emission scenarios [37]. The probability of sustained malaria transmission occurring in the UK in the current century was found to be small, even under the most extreme emission scenario.

It is important to note, however, that all five malaria models were developed and parameterized for malaria caused by *P. falciparum*, while it was malaria from *P. vivax* that was previously endemic in the UK and which has recently reoccurred in Europe. *Plasmodium vivax* transmission by European vectors requires lower temperature thresholds than *P. falciparum* and, therefore, all five malaria models and WHO's assessments underestimate the risk of malaria in Europe. By contrast, under a medium-high scenario of climate change, a model parameterized for *P. vivax* predicts that the southern half of Great Britain will be climatically-suitable for *P. vivax* malaria transmission by 2030 for 2 months of the year, and parts of the southeast of England for 4 months per year [38]; while by 2080 even southern Scotland will be climatically-suitable for 2 months of the year [39].

The risk of malaria transmission is, however, determined by many factors other than climate; and while climate change may increase the suitability of the UK for transmission, other factors (such as drainage of marshes and changes in land use) make the probability of it occurring small.

The main threat to the UK from dengue, chikungunya and Zika viruses is tied to the risk of invasion by *Ae. aegypti* and *Ae. albopictus*. The suitability of the current European climate for both vectors and dengue transmission has been modelled [40]. Much of Europe, including many parts of the UK, were found to be suitable for *Ae. albopictus*; however, few areas of Europe, and none in the UK, were suitable for *Ae. aegypti*. Other workers report similar findings [41]. Three models indicate that large parts of the UK are already suitable for *Ae. albopictus* and predict increased climatic suitability in future.

Arbovirus transmission requires that environmental conditions are suitable for both virus and vector, and even where vectors are present, it may be too cold for viral transmission. Studies of the climatic needs of chikungunya virus (based on conditions at past outbreaks) indicate a threshold minimum temperature of 20-22°C. Combining this threshold with the climatic needs of the vector, the UK is found to be climatically unsuitable for chikungunya transmission at the present time [42] although some parts of England may become 'rather unsuitable' (the second lowest

of five risk classes) later in the century. No parts of the UK become suitable for dengue virus development (in *Aedes aegypti*) by 2100 [43].

While there is consensus that climate affects the transmission of tick-borne Lyme borreliosis, and some agreement that the northward spread of the vector in Sweden is attributable to recent warming, there are no published models projecting its future incidence in Europe under scenarios of climate change. Models for other temperate regions predict that climate change will allow northern expansion of the range of the tick vectors [44]. Lyme and its vectors are already found throughout the UK and, therefore, increasing climate suitability may be limited to increase in altitude [45] and change in incidence. Non-climate drivers, however, play a dominant role in the epidemiology of Lyme disease [46] and future trends in agriculture, land use, wild animal populations and tourism will play a large role in determining future patterns of the disease [47].

Potential for impacts to be avoided by adaptation measures

Adaptation to the risk of emerging infections requires three actions: (i) improved surveillance of disease and, where appropriate, vectors, so that sporadic cases and outbreaks, or the heightened risk of these, can be detected early; (ii) risk assessments of the likelihood of cases or outbreaks of an emerging disease, regularly updated as new data become available; and (iii) increased preparedness for outbreaks.

The northward spread of *Ae. albopictus* indicates the need for active surveillance for invasive mosquitoes. There is strong evidence that some of the spread of *Ae. albopictus* in continental Europe is associated with transport in vehicles as, for example, it has been trapped in motorway service areas. The extensive movement of cars and goods vehicles into the UK from continental Europe should be considered a likely route of entry of this mosquito. Insecticidal treatment of vehicles entering the UK could help to reduce this risk. 'Disinsection', as described by the International Health Regulations of the WHO, is already used to kill insects in airplanes arriving in certain countries. *Aedes albopictus* has also entered many countries in used vehicle tyres or in 'lucky bamboo', a houseplant imported from China. Mosquito surveillance at ports of entry of such products is recommended.

A known vector of West Nile virus, *Culex modestus*, has re-established in the UK. Surveillance within affected regions can help identify areas of particular risk of virus transmission; and surveillance outside such areas can be used to monitor its further spread.

Adaptation measures to Lyme disease include increasing awareness of the public, in terms of recognizing symptoms and taking tick-avoidance measures; and improving national-level surveillance. For surveillance, it has been recommended to base the clinical definition of Lyme disease on the skin rash that occurs in about 90% of cases, rather than on the rare Lyme neuroborreliosis that needs laboratory confirmation [3]. Forecasts of the risk of exposure to tick vectors or Lyme, based on geographic and climatic information, will help the public to take suitable precautions.

Comment [JS10]: Lyme disease is currently being considered for mandatory reporting in the EU.

The risk of emerging disease outbreaks in the UK, particularly vector-borne, food-borne and water-borne diseases, is likely to continue to rise as our climate changes. New data will frequently become available, such as updated maps of the occurrence of potentially invasive vectors in continental Europe. Risk assessments should, therefore, be regularly updated.

Preparedness for outbreaks of vector-borne diseases requires (i) knowledge of the vectors, (ii) identification of areas of high risk, (iii) in such areas, raised awareness of the public and health professionals and (iv) development of policy and approval of legislation to permit vector control programmes. A contingency plan should be developed for the event that *Ae. albopictus* is detected in the UK.

Conclusions

Given the sensitivity of many diseases to climate, it is very likely that at least some will respond to future climate change. Many water-borne and food-borne diseases are climate sensitive but there is a relative paucity of published studies on the impacts of climate change upon them. The impact of climate change on infectious respiratory diseases requires further study, as such diseases can spread rapidly and cause significant health impact; detailed studies of the impacts of climate change on them, however, has received little attention [48].

This review has focused extensively on vector-borne diseases because they are particularly climate-sensitive. Several vector-borne diseases have recently emerged in Europe, and some threaten the UK. The greatest risk is probably presented by West Nile virus, as is widespread in southern and eastern Europe and the UK harbours at least one competent vector species. There is a threat of invasion by the Asian Tiger mosquito, *Ae. albopictus*; if the vector enters and survives in the UK, however, it may be too cold for sustained transmission of dengue, chikungunya or Zika viruses. The UK is, and long has been, climatically suitable for the transmission of malaria caused by *P. vivax*. Climate change will increase the climatic suitability even further, but this is unlikely to lead to sustained malaria transmission if land use and agricultural practices remain as they are today. However, the recreation of extensive wetlands and salt marshes, combined with warmer temperatures, may create a situation where local vivax malaria transmission is possible.

The emergence of Lyme disease in the UK is likely to continue in the future. Climate change may contribute to this emergence, but other drivers will probably play a larger role. The risk presented by TBE, which is emerging in many parts of Europe, remains difficult to gauge.

Key research gaps are: (i) assessment of the impact of climate change on diseases that are not vector-borne, including respiratory infections; (ii) evaluation of the vector potential of UK native species; (iii) development of climate driven models for the risk of West Nile and Lyme in the UK; (iv) evaluation of the threat posed by TBE to the UK and (v) incorporation of environmental and agricultural variables into models of the risk of vivax malaria in the UK.

Comment [JS11]: And other variables. Indeed, the incorporation of other sociodemographic variables into these models remains one of the technical challenges of the field.

Competing interests

The author declares there are no competing interests

DIsclaimer

The views expressed are those of the author(s) and not necessarily those of the NHS, the NIHR, the Department of Health or Public Health England.

References

1. Morens DM, Folkers GK, Fauci AS: The challenge of emerging and re-emerging infectious diseases. *Nature* 2004, 430(6996):242-249.
2. McIntyre KM, Setzkorn C, Hepworth PJ, Morand S, Caminade C, Morse AP *et al*: Risk assessment of climate-change impacts on infectious diseases in Europe. *Nature Climate Change* 2016, submitted.
3. Lindgren E, Andersson Y, Suk JE, Sudre B, Semenza JC: Monitoring EU Emerging Infectious Disease Risk Due to Climate Change. *Science* 2012, 336(6080):418-419.
4. Cann K, Thomas DR, Salmon R, Wyn-Jones A, Kay D: Extreme water-related weather events and waterborne disease. *Epidemiol Infect* 2013, 141(04):671-686.
5. Eze J, Scott E, Pollock K, Stidson R, Miller C, Lee D: The association of weather and bathing water quality on the incidence of gastrointestinal illness in the west of Scotland. *Epidemiol Infect* 2014, 142(06):1289-1299.
6. Jones KE, Patel NG, Levy MA, Storeygard A, Balk D, Gittleman JL *et al*: Global trends in emerging infectious diseases. *Nature* 2008, 451(7181):990-993.
7. Kuhn KG, Campbell-Lendrum DH, Armstrong B, Davies CR: Malaria in Britain: Past, present, and future. *Proc Natl Acad Sci U S A* 2003, 100(17):9997-10001.
8. Danis K, Baka A, Lenglet A, Van Bortel W, Terzaki I, Tseroni M *et al*: Autochthonous Plasmodium vivax malaria in Greece, 2011. *Eurosurveillance* 2011, 16(42).
9. EFSA/ECDC: The European Union Summary Report on Trends and Sources of Zoonoses, Zoonotic Agents and Food-borne Outbreaks in 2012. *EFSA Journal* 2014, 12(2):1-312.
10. Morin CW, Comrie AC: Regional and seasonal response of a West Nile virus vector to climate change. *Proc Natl Acad Sci U S A* 2013, 110(39):15620-15625.
11. Pradier S, Lecollinet S, Leblond A: West Nile virus epidemiology and factors triggering change in its distribution in Europe. *Rev Sci Tech OIE* 2012, 31(3):829-844.
12. Medlock JM, Vaux AGC: Distribution of West Nile virus vector, Culex modestus, in England. *Vet Rec* 2012, 171(11).
13. Parker DE, Legg TP, Folland CK: A New Daily Central England Temperature Series, 1772-1991. *Int J Climatol* 1992, 12(4):317-342.

14. Mellor PS, Carpenter S, Harrup L, Baylis M, Mertens PPC: Bluetongue in Europe and the Mediterranean Basin: History of occurrence prior to 2006. *Prev Vet Med* 2008, 87(1-2):4-20.
15. Mackenzie-Impoinvil L, Impoinvil DE, Galbraith SE, Dillon RJ, Ranson H, Johnson N *et al*: Evaluation of a temperate climate mosquito, Ochlerotatus detritus (= Aedes detritus), as a potential vector of Japanese encephalitis virus. *Med Vet Entomol* 2015, 29(1):1-9.
16. Angelini R, Finarelli AC, Angelini P, Po C, Petropulacos K, Silvi G *et al*: Chikungunya in north-eastern Italy: a summing up of the outbreak. *Eurosurveillance* 2007, 12(47).
17. Grandadam M, Caro V, Plumet S, Thiberge J-M, Souares Y, Failloux A-B *et al*: Chikungunya Virus, Southeastern France. *Emerg Infect Dis* 2011, 17(5):910-913.
18. La Ruche G, Souares Y, Armengaud A, Peloux-Petiot F, Delaunay P, Despres P *et al*: First two autochthonous dengue virus infections in metropolitan France, September 2010. *Eurosurveillance* 2010, 15(39):2-6.
19. Gjenero-Margan I, Aleraj B, Krajcar D, Lesnikar V, Klobucar A, Pem-Novosel I *et al*: Autochthonous dengue fever in Croatia, August-September 2010. *Eurosurveillance* 2011, 16(9):2-5.
20. Marchand E PC, Jeannin C, Lafont E, Bergmann T, Flusin O, Rizzi J, Roux N, Busso V, Deniau J, Noel H, Vaillant V, Leparc-Goffart I, Six C, Paty MC: Autochthonous case of dengue in France, October 2013. *Eurosurveillance* 2013, 18(50).
21. Almeida A, Gonçalves Y, Novo M, Sousa C, Melim M, AJ G: Vector monitoring of Aedes aegypti in the Autonomous Region of Madeira, Portugal. *Eurosurveillance* 2007, 12(46).
22. Sousa CA, Clairouin M, Seixas G, Viveiros B, Novo MT, Silva AC *et al*: Ongoing outbreak of dengue type 1 in the Autonomous Region of Madeira, Portugal: preliminary report. *Eurosurveillance* 2012, 17(49):15-18.
23. Chouin-Carneiro T, Vega-Rua A, Vazeille M, Yebakima A, Girod R, Goindin D *et al*: Differential Susceptibilities of Aedes aegypti and Aedes albopictus from the Americas to Zika Virus. *PLoS Negl Trop Dis* 2016, 10(3):e0004543.
24. Foy BD, Kobylinski KC, Chilson Foy JL, Blitvich BJ, Travassos da Rosa A, Haddow AD *et al*: Probable non-vector-borne transmission of Zika virus, Colorado, USA. *Emerg Infect Dis* 2011, 17(5):880-882.
25. Dujardin JC, Campino L, Canavate C, Dedet JP, Gradoni L, Soteriadou K *et al*: Spread of vector-borne diseases and neglect of leishmaniasis, Europe. *Emerg Infect Dis* 2008, 14(7):1013-1018.
26. Ready PD: Leishmaniasis emergence in Europe. *Eurosurveillance* 2010, 15(10):29-39.
27. Shaw SE, Langton DA, Hillman TJ: Canine leishmaniosis in the United Kingdom: A zoonotic disease waiting for a vector? *Vet Parasitol* 2009, 163(4):281-285.
28. Stefanoff P, Parda N, Polkowska A, D'Anconca F, Anda P, Rizzoli A *et al*: Epidemiological situation of tick-borne encephalitis in the European Union and European Free Trade Association countries. In: *ECDC Technical Report*. Stockholm: European Centre for Disease Prevention and Control; 2012.

29. Randolph SE: Evidence that climate change has caused 'emergence' of tick-borne diseases in Europe? *Int J Med Microbiol* 2004, 293:5-15.
30. Sumilo D, Asokliene L, Bormane A, Vasilenko V, Golovljova I, Randolph SE: Climate Change Cannot Explain the Upsurge of Tick-Borne Encephalitis in the Baltics. *PLoS One* 2007, 2(6).
31. Lindgren E, Talleklint L, Polfeldt T: Impact of climatic change on the northern latitude limit and population density of the disease-transmitting European tick Ixodes ricinus. *Environ Health Perspect* 2000, 108(2):119-123.
32. Maltezou HC, Andonova L, Andraghetti R, Bouloy M, Ergonul O, Jongejan F *et al*: Crimean-Congo hemorrhagic fever in Europe: current situation calls for preparedness. *Eurosurveillance* 2010, 15(10):48-51.
33. Jameson LJ, Morgan PJ, Medlock JM, Watola G, Vaux AGC: Importation of Hyalomma marginatum, vector of Crimean-Congo haemorrhagic fever virus, into the United Kingdom by migratory birds. *Ticks Tick Borne Dis* 2012, 3(2):95-99.
34. Atkinson B, Latham J, Chamberlain J, Logue C, O'Donoghue L, Osborne J *et al*: Sequencing and phylogenetic characterisation of a fatal Crimean - Congo haemorrhagic fever case imported into the United Kingdom, October 2012. *Eurosurveillance* 2012, 17(48):7-10.
35. McMichael AJ, Campbell-Lendrum D, Kovats S, Edwards SJ, Wilkinson P, Wilson T *et al*: Global Climate Change. Global and Regional Burden of Disease Attributable to Selected Major Risk Factors. In: *Comparative Quantification of Health Risks: Global and Regional Burden of Disease Attributable to Selected Major Risk Factors.* Edited by Ezzati M, Lopez AD, Rodgers A, Murray CJL, vol. 2. Geneva: World Health Organization; 2004: 1543-1649.
36. Hales S, Kovats S, Lloyd S, Campbell-Lendrum D (eds.): Quantitative risk assessment of the effects of climate change on selected causes of death, 2030s and 2050s. Geneva: World Health Organisation; 2014.
37. Caminade C, Kovats S, Rocklov J, Tompkins AM, Morse AP, Colón-González FJ *et al*: Impact of climate change on global malaria distribution. *Proc Natl Acad Sci U S A* 2014, 111(9):3286-3291.
38. Lindsay SW, Hole DG, Hutchinson RA, Richards SA, Willis SG: Assessing the future threat from vivax malaria in the United Kingdom using two markedly different modelling approaches. *Malar J* 2010, 9:70.
39. Lindsay SW, Thomas CJ: Global warming and risk of vivax malaria in Great Britain. *Global Change and Human Health* 2001, 2(1):80-84.
40. Rogers D, Hay SI: The climatic suitability for dengue transmission in continental Europe. In: *ECDC Technical Report.* Stockholm: European Centre for Disease Prevention and Control; 2012: 1-22.
41. Caminade C, Medlock JM, Ducheyne E, McIntyre KM, Leach S, Baylis M *et al*: Suitability of European climate for the Asian tiger mosquito Aedes albopictus: recent trends and future scenarios. *J R Soc Interface* 2012, 9(75):2708-2717.
42. Fischer D, Thomas SM, Suk JE, Sudre B, Hess A, Tjaden NB *et al*: Climate change effects on Chikungunya transmission in Europe: geospatial analysis of vector's climatic suitability and virus' temperature requirements. *Int J Health Geogr* 2013, 12.

43. Thomas SM, Fischer D, Fleischmann S, Bittner T, Beierkuhnlein C: Risk assessment of dengue virus amplification in europe based on spatio-temporal high resolution climate change projections. *Erdkunde* 2011, 65(2):137-150.
44. Ogden NH, Maarouf A, Barker IK, Bigras-Poulin M, Lindsay LR, Morshed MG *et al*: Climate change and the potential for range expansion of the Lyme disease vector Ixodes scapularis in Canada. *Int J Parasitol* 2006, 36(1):63-70.
45. Gilbert L: Altitudinal patterns of tick and host abundance: a potential role for climate change in regulating tick-borne diseases? *Oecologia* 2010, 162(1):217-225.
46. Rizzoli A, Hauffe HC, Carpi G, Vourc'h GI, Neteler M, Rosa R: Lyme borreliosis in Europe. *Eurosurveillance* 2011, 16(27):2-9.
47. Lindgren E, Jaenson TGT: Lyme borreliosis in Europe: influences of climate and climate change, epidemiology, ecology and adaption measures. In. Geneva: World Health Organization; 2006.
48. Ayres J, Forsberg B, Annesi-Maesano I, Dey R, Ebi K, Helms P *et al*: Climate change and respiratory disease: European Respiratory Society position statement. *Eur Respir J* 2009, 34(2):295-302.

Reviewer 2: Joacim Rocklov

Competing interests declaration: I declare that I have no competing interests.

Potential impact of climate change on emerging infections in the UK

Matthew Baylis

Liverpool University Climate and Infectious Diseases of Animals group, Institute of Infection and Global Health, University of Liverpool; & Health Protection Research Unit in Emerging and Zoonotic Infections, University of Liverpool.

matthew.baylis@liv.ac.uk

Abstract

Climate is one of several causes of disease emergence. Although half or more of infectious diseases are affected by climate it appears to be a relatively infrequent cause of human disease emergence. Climate mostly affects diseases caused by pathogens that spend part of their lifecycle outside of the host, exposed to the environment. The most important routes of transmission of climate sensitive diseases are by arthropod (insect and tick) vectors, in water and in food. Given the sensitivity of many diseases to climate, it is very likely that at least some will respond to future climate change. In the case of vector-borne diseases this response will include spread to new areas. Several vector-borne diseases have emerged in Europe in recent years; these include vivax malaria, West Nile fever, dengue fever, Chikungunya fever, leishmaniasis, Lyme disease and tick-borne encephalitis. The vectors of these diseases are mosquitoes, sand flies and ticks. The UK has endemic mosquito species capable of transmitting malaria and probably other pathogens, and ticks that transmit Lyme disease. The UK is also threatened by invasive mosquito species known to be able to transmit West Nile, dengue, chikungunya and Zika, and sand flies that spread leishmaniasis. Warmer temperatures in the future will increase the suitability of the UK's climate for these invasive species, and increase the risk that they may spread disease. While much attention is on invasive species, it is important to recognize the threat presented by native species too. Proposed actions to reduce the future impact of emerging vector-borne diseases in the UK include insect control activity at points of entry of vehicles and certain goods, wider surveillance for mosquitoes and sand flies, research into the threat posed by native species, increased awareness of the medical profession of the threat posed by specific diseases, regular risk assessments, and increased preparedness for the occurrence of a disease emergency.

Keywords

Climate; Climate change; disease; vector; mosquito; tick; sandfly, West Nile, Lyme, malaria, Zika

Comment [JR1]: The work reflects Europe largely and the title appear a bit misleading unless Britain has expansion plans in the near future… I would recommend considering re-writing the title.

Maybe add " vector-borne" as most of the more indepth content is discussing vector-borne diseases?

Introduction

There is widespread scientific agreement that anthropogenic activities are contributing to the warming of the world's climate at an unprecedented rate, with concomitant changes in precipitation, flooding, winds and the frequency of extreme events. Many studies have demonstrated links between infectious diseases and climate, and it is highly likely that changes in climate will impact on at least some of them. This review summarises the state of scientific evidence regarding current and future impacts of anthropogenic climate change on the emergence of infectious diseases with potential to impact significantly on the UK human population. Infections are defined as 'emerging' if they "have newly appeared in a population or have existed previously but are rapidly increasing in incidence or geographic range" [1]. Initially the review considers a broad range of possible emerging infections but then narrows the focus to vector-borne diseases.

Emerging diseases

Human diseases are affected by climate when the pathogen spends a significant period of time outside of the human host, subject to environmental influence. The most climate-sensitive diseases are, therefore, those spread by arthropod vectors, in food or water, in aerosol, in fomites, and those with free-living stages.

By contrast, most diseases transmitted directly between humans (for example, human childhood viruses, sexually transmitted diseases, tuberculosis) have few or no reported associations with climate. Exceptions are the human respiratory viruses, such as those that cause colds and seasonal flu, which spread by aerosol between individuals in close contact but are nevertheless sensitive to the effects of ambient humidity and possibly temperature, and have strong seasonal cycles.

Systematic reviews of disease-climate associations support these assertions. Scrutiny of research papers on 157 high-impact human and animal pathogens of Europe found that 66% have at least one climate variable that affects disease occurrence. Transmission routes of climate sensitive diseases, in decreasing order of importance, were vectors, food/water, environment, fomite and aerosol [2]. Similarly, a weighted risk analysis following systematic review identified 26 (49%) out of 53 reportable infectious diseases of humans in Europe to be directly or indirectly linked to climate [3].

Climate affects three major aspects of disease occurrence: where, when and how much. In some cases, mostly macroparasite infections caused by helminths, climate can also affect the severity of clinical disease in infected individuals.

Vector-borne diseases usually have geographically-restricted distributions because of the effects of climate of the insects/ticks. Such diseases are particularly likely, therefore, to spread to new areas with climate change. Some water-borne diseases

may also spread in response to climate change, while the response of other climate-sensitive diseases may be change to their seasonal cycles or the frequency or scale of disease outbreaks in endemic regions.

Extreme weather events are an important factor in the spread of water-borne diseases such as cholera, leptospirosis and gastrointestinal infections. A systematic review [4] found that heavy rainfall and flooding frequently precede water-borne disease outbreaks. At the other extreme, drought can lead to the concentration of water-borne pathogens in rivers and water bodies. Temperature is also important: higher temperatures lead to faster growth rates of disease-causing pathogens, and increased use of bathing waters by the public [5].

Climate change is just one of several forces ("drivers") that may lead to the emergence of infectious diseases. Other drivers are changes to the environment through deforestation and urbanization; developments in agriculture and food production; changes in how people live, behave, eat, travel and trade; changes in medicine, public health and the use of antimicrobials; and the occurrence of "shocks" such as war, migration and famine [6]. An analysis of more than 300 human disease outbreaks concluded that climate and weather were infrequent causes (3%). Some other drivers, however, such as changes to land use and agriculture (11%), are themselves affected by climate change. Therefore, climate change's indirect effects on disease emergence (via effects on other drivers) may also be important.

There are, therefore, strong arguments to expect climate change to impact on human disease outbreaks, especially those transmitted by vectors or in food/water, but in practice there are relatively few examples. Recently, however, Europe has seen an upsurge in vector-borne diseases, as well as the spread of vectors, and the possible role of climate change needs consideration.

Recent impact of climate change on vector-borne disease emergence in Europe

The main vectors threatening or affecting the health of people in Europe are mosquitoes, sandflies and ticks.

Malaria, caused by *Plasmodium vivax* and spread by the bites of *Anopheles* mosquitoes, used to be endemic the UK but there has been no transmission via native mosquitoes for over 60 years [7]. The disappearance of malaria is attributed largely to changes to the environment (drainage of marshes) and farming (Kuhn et al., 2003). Similar changes have occurred in mainland Europe and no areas are now endemic for the disease. In 2011, however, an outbreak of *P. vivax* malaria occurred in Greece, after nearly 40 years of only sporadic cases . This is not linked to climate change: the origin is likely to be migrant workers introducing *P. vivax* into an agricultural region where vectors were present [8].

West Nile fever, caused by West Nile virus (WNV) and spread by *Culex* mosquitoes, is endemic in a number of southern and eastern European countries. The incidence of human cases is increasing [9]. WNV vectors are very sensitive to changes in temperature [10], and increasing temperature is considered to be a driver of its emergence in Europe [11]. In the UK there have been only unconfirmed reports of the transmission of WNV among birds. However, a known WNV vector, *Culex*

modestus, was recently rediscovered in southern England, after no reports in the UK since the 1940s [12]. This re-establishment has not been linked to climate change, but it is interesting to note that the 1940s were the warmest recorded period in England (Central England time series) until the present [13].

Indigenous UK mosquito species may present an underestimated risk for the transmission of arboviruses. For a mosquito to act as a vector, it needs to live longer than the time required for a pathogen to develop within it, and native species may be more able to survive (and hence live longer) at the UK's relatively cool temperatures than exotic species. There is a good example from the field of animal disease: bluetongue, a midge-borne viral disease of ruminants, is transmitted in Mediterranean countries by an invasive, African midge species. It was not foreseen that indigenous midges could transmit bluetongue in climates as cool as southern Scandinavia. In 2006-2009, tens of thousands of farms across northern Europe, including some in the UK, were affected by this disease [14]. It is noteworthy, therefore, that a UK native mosquito, *Ochleratatus detritus,* which feeds readily on humans, is a good vector of flaviviruses, the group that includes WNV [15].

Invasive mosquitoes also present a risk of the introduction of new diseases. There have been several outbreaks of Chikungunya fever and dengue in north Italy, southern France and Croatia since 2007 [16-20]. The vector is the Asian tiger mosquito, *Aedes albopictus*, an invasive species that has spread from Asia to North America and Europe, where it is now found in most southern European countries but also Belgium and The Netherlands. The major dengue vector, *Ae. aegypti*, disappeared from Europe in the mid-20th century but has now returned, being found in Madeira (an autonomous region of Portugal) in 2004-05 [21]. Despite vector control efforts, the mosquito has thrived; and in 2012 there were more than 2,000 dengue cases, the first sustained transmission of dengue in Europe since the 1920s [22].

The emergence of dengue and Chikungunya in Europe is associated with the establishment of *Ae. albopictus* and *Ae. aegypti*, together with mutation of Chikungunya virus that facilitated infection of *Ae. albopictus*. The mosquitoes became infected after feeding on returning travellers carrying infections obtained overseas. The emergence is, therefore, not associated with climate change.

There is a significant risk that Zika virus, which is believed to have infected over a million people in South America in 2015-2016, will emerge in Europe in 2016. Both *Ae. aegypti* and *Ae. albopictus* are competent vectors [23] and the greatest risk is where these vectors are present. However, Zika virus can also be spread sexually [24], and there is a small risk of transmission outside of areas with competent vectors.

Sandflies are vectors of cutaneous and visceral leishmaniasis in people and dogs in southern Europe. There is a low incidence in people (~700 cases per year in southern Europe) but a high incidence in dogs (about 5,000 cases per year in France alone) [25]. Sandflies have not been detected in the UK but have been found in central and northern France and southern Germany [26]. The presence of known vectors in northern Europe, and the passport-controlled movement of potentially infected

Comment [JR2]: Appear vectors are expanding their territory in Europe

Comment [JR3]: In my view travellers would need to carry virus with or without climate change being the reasons to emergence. The climate question is probably more whether climate change, or anomalies the outbreak years, explain vector establishment or greater abundance of vectors and their vectorial capacity. Therefore, claiming it is not associated with climate change is rather strong as climate change may well have increased the probability for these events, even if only very marginally. I would agree if the statement was more towards travelling rather than climate change was the main reason for emergence/re-emergence.

Comment [JR4]: Or the next couple of years (since no transmission actually have happened in 2016 in Europe.)

Comment [JR5]: Few new studies show a lower competence of European albopictus to Zika basically limiting this risk (if these studies are correct). But both vectors appear competent.

Comment [JR6]: There is a paper by Yakobs et all on this in the Lancet that may support " small" risk.

dogs into the UK [27], raises the possibility of the eventual introduction of leishmaniasis.

Two tick-borne diseases have emerged in Europe in recent decades, tick-borne encephalitis (TBE) and Lyme disease, both transmitted by the sheep tick, *Ixodes ricinus.* The emergence of TBE is a major concern [28]. There are thousands of cases of human disease each year, the incidence is increasing, and it is spreading to new regions. Possible causes of this emergence are socioeconomic, environmental and climatic changes, as well as increased awareness (Stefanoff, 2012 #3038; Lukan, 2010 #3026}. The role of climate change is contested [29] and other drivers of disease emergence, such as change in human behavior, believed to be important [30].

While TBE occurs as far north as Scandinavia (indicating that the UK climate should not be a barrier to disease), and the *I. ricinus* vector is found throughout the British Isles, TBE virus has not been reported in the UK. This may be because *I. ricinus* transmits the related Louping ill virus in the UK, which causes disease in sheep and only rarely affects people.

A bacterial spirochaete disease, called Lyme borreliosis and also spread by *I. ricinus*, is emerging in the UK. Lyme disease is the most common vector-borne disease in temperate climates, with an estimated 85,000 cases per year in Europe; over 2,000 cases occur per year in the UK alone. Lyme disease is increasing in incidence, possibly because of climate-change influences on the tick vector. Increased *I. ricinus* density and its spread to higher latitude in Sweden have been linked to milder winters [31].

Tick-borne disease threats also include Crimean Congo Haemorrhagic Fever (CCHF). This viral disease is spread largely by *Hyalomma* ticks, is endemic in parts of Eastern and south-eastern Europe; and it appears to be emerging [32]. *Hyalomma* ticks are not endemic to the UK although they may arrive on migrant birds [33]; and CCHF virus may arrive in infected travellers [34].

Most consideration has been given here to vector-borne diseases but it is important to note that many non-vector-borne diseases have emerged in recent years. Of 38 human pathogens listed by Public Health England as having emerged (globally) since 1980, only 4 are vector-borne. The emergence of 25 of these 38 pathogens is included in a study of drivers of emergence [6] and none were attributed to climate. In other words, the majority of emerging diseases are not vector-borne, and their emergence is not linked to climate change.

Future impact of climate change on vector-borne disease emergence in Europe

The World Health Organization has attempted to quantify the additional amount of human disease that might arise as a consequence of climate change [35, 36]. Inevitably, there is significant uncertainty. In a recent assessment for the years 2030 and 2050, WHO found increases of 20 - 86,000 deaths globally from diarrhoeal disease of children attributable to climate change, under a range of socioeconomic growth scenarios [36]. However, the number in Western Europe was very small (1 or

2). Equally, climate change led to no projected deaths in Western Europe from *P. falciparum* malaria or dengue.

Several other workers have developed models of climate change's future impacts on malaria. The most extensive examined the projections of five malaria impact models, each driven by five global climate models and four emission scenarios [37]. The probability of sustained malaria transmission occurring in the UK in the current century was found to be small, even under the most extreme emission scenario.

It is important to note, however, that all five malaria models were developed and parameterized for malaria caused by *P. falciparum*, while it was malaria from *P. vivax* that was previously endemic in the UK and which has recently reoccurred in Europe. *Plasmodium vivax* transmission by European vectors requires lower temperature thresholds than *P. falciparum* and, therefore, all five malaria models and WHO's assessments underestimate the risk of malaria in Europe. By contrast, under a medium-high scenario of climate change, a model parameterized for *P. vivax* predicts that the southern half of Great Britain will be climatically-suitable for *P. vivax* malaria transmission by 2030 for 2 months of the year, and parts of the southeast of England for 4 months per year [38]; while by 2080 even southern Scotland will be climatically-suitable for 2 months of the year [39].

The risk of malaria transmission is, however, determined by many factors other than climate; and while climate change may increase the suitability of the UK for transmission, other factors (such as drainage of marshes and changes in land use) make the probability of it occurring small.

The main threat to the UK from dengue, chikungunya and Zika viruses is tied to the risk of invasion by *Ae. aegypti* and *Ae. albopictus*. The suitability of the current European climate for both vectors and dengue transmission has been modelled [40]. Much of Europe, including many parts of the UK, were found to be suitable for *Ae. albopictus*; however, few areas of Europe, and none in the UK, were suitable for *Ae. aegypti*. Other workers report similar findings [41]. Three models indicate that large parts of the UK are already suitable for *Ae. albopictus* and predict increased climatic suitability in future.

Arbovirus transmission requires that environmental conditions are suitable for both virus and vector, and even where vectors are present, it may be too cold for viral transmission. Studies of the climatic needs of chikungunya virus (based on conditions at past outbreaks) indicate a threshold minimum temperature of 20-22°C. Combining this threshold with the climatic needs of the vector, the UK is found to be climatically unsuitable for chikungunya transmission at the present time [42] although some parts of England may become 'rather unsuitable' (the second lowest of five risk classes) later in the century. No parts of the UK become suitable for dengue virus development (in *Aedes aegypti*) by 2100 [43].

While there is consensus that climate affects the transmission of tick-borne Lyme borreliosis, and some agreement that the northward spread of the vector in Sweden is attributable to recent warming, there are no published models projecting its future incidence in Europe under scenarios of climate change. Models for other temperate regions predict that climate change will allow northern expansion of the range of the tick vectors [44]. Lyme and its vectors are already found throughout the

Comment [JR7]: It may be worthwhile to explain that Vivax has been transmitted as far north as Sweden in the beginning of the 20th century, and that its eradication/disappearance can be highly attributed to environmental, socio-economic changes. Maybe also worthwhile to note that malaria is currently transmitted in European airports (e.g Hay et al)

Comment [JR8]: Studies suggest, however, the socio-economic conditions can make big difference in transmission risk (e.g. P Reiter Mexico/US border study)

Comment [JR9]:

Zika:
Rocklov J, Quam MB, Sudre B, German M, Kraemer MU, Brady O, et al. Assessing Seasonal Risks for the Introduction and Mosquito-borne Spread of Zika Virus in Europe. EBioMedicine. 2016;9:250-6.

Dengue Aegypti and Albopictus:
Liu-Helmersson J. QMB, Wilder-Smith A., Stenlund H., Ebi K., Massad E., Rocklöv J. . Climate Change and Aedes Vectors: 21st Century Projections for Dengue Transmission in Europe. eBioMedicine. 2016.

Dengue Aegypti:
Liu-Helmersson J, Stenlund H, Wilder-Smith A, Rocklov J. Vectorial capacity of Aedes aegypti: effects of temperature and implications for global dengue epidemic potential. PloS one. 2014;9(3):e89783.

UK and, therefore, increasing climate suitability may be limited to increase in altitude [45] and change in incidence. Non-climate drivers, however, play a dominant role in the epidemiology of Lyme disease [46] and future trends in agriculture, land use, wild animal populations and tourism will play a large role in determining future patterns of the disease [47].

Potential for impacts to be avoided by adaptation measures

Adaptation to the risk of emerging infections requires three actions: (i) improved surveillance of disease and, where appropriate, vectors, so that sporadic cases and outbreaks, or the heightened risk of these, can be detected early; (ii) risk assessments of the likelihood of cases or outbreaks of an emerging disease, regularly updated as new data become available; and (iii) increased preparedness for outbreaks.

Comment [JR10]: I would strongly suggest forecast/early warning systems as an adaptation measure to inform timeliness and spatial extent of preparedness and control. Such may early warning, for example, build on weather, mobility, vector surveillance and disease transmission in non-European or neighboring regions.

The northward spread of *Ae. albopictus* indicates the need for active surveillance for invasive mosquitoes. There is strong evidence that some of the spread of *Ae. albopictus* in continental Europe is associated with transport in vehicles as, for example, it has been trapped in motorway service areas. The extensive movement of cars and goods vehicles into the UK from continental Europe should be considered a likely route of entry of this mosquito. Insecticidal treatment of vehicles entering the UK could help to reduce this risk. 'Disinsection', as described by the International Health Regulations of the WHO, is already used to kill insects in airplanes arriving in certain countries. *Aedes albopictus* has also entered many countries in used vehicle tyres or in 'lucky bamboo', a houseplant imported from China. Mosquito surveillance at ports of entry of such products is recommended.

A known vector of West Nile virus, *Culex modestus*, has re-established in the UK. Surveillance within affected regions can help identify areas of particular risk of virus transmission; and surveillance outside such areas can be used to monitor its further spread.

Adaptation measures to Lyme disease include increasing awareness of the public, in terms of recognizing symptoms and taking tick-avoidance measures; and improving national-level surveillance. For surveillance, it has been recommended to base the clinical definition of Lyme disease on the skin rash that occurs in about 90% of cases, rather than on the rare Lyme neuroborreliosis that needs laboratory confirmation [3]. Forecasts of the risk of exposure to tick vectors or Lyme, based on geographic and climatic information, will help the public to take suitable precautions.

The risk of emerging disease outbreaks in the UK, particularly vector-borne, food-borne and water-borne diseases, is likely to continue to rise as our climate changes. New data will frequently become available, such as updated maps of the occurrence of potentially invasive vectors in continental Europe. Risk assessments should, therefore, be regularly updated.

Preparedness for outbreaks of vector-borne diseases requires (i) knowledge of the vectors, (ii) identification of areas of high risk, (iii) in such areas, raised awareness of the public and health professionals and (iv) development of policy and approval of

legislation to permit vector control programmes. A contingency plan should be developed for the event that *Ae. albopictus* is detected in the UK.

Conclusions

Given the sensitivity of many diseases to climate, it is very likely that at least some will respond to future climate change. Many water-borne and food-borne diseases are climate sensitive but there is a relative paucity of published studies on the impacts of climate change upon them. The impact of climate change on infectious respiratory diseases requires further study, as such diseases can spread rapidly and cause significant health impact; detailed studies of the impacts of climate change on them, however, has received little attention [48].

Comment [JR11]: I feel this could be discuss more indepth prior to conclusion

This review has focused extensively on vector-borne diseases because they are particularly climate-sensitive. Several vector-borne diseases have recently emerged in Europe, and some threaten the UK. The greatest risk is probably presented by West Nile virus, as is widespread in southern and eastern Europe and the UK harbours at least one competent vector species. There is a threat of invasion by the Asian Tiger mosquito, *Ae. albopictus*; if the vector enters and survives in the UK, however, it may be too cold for sustained transmission of dengue, chikungunya or Zika viruses. The UK is, and long has been, climatically suitable for the transmission of malaria caused by *P. vivax*. Climate change will increase the climatic suitability even further, but this is unlikely to lead to sustained malaria transmission if land use and agricultural practices remain as they are today. However, the recreation of extensive wetlands and salt marshes, combined with warmer temperatures, may create a situation where local vivax malaria transmission is possible.

The emergence of Lyme disease in the UK is likely to continue in the future. Climate change may contribute to this emergence, but other drivers will probably play a larger role. The risk presented by TBE, which is emerging in many parts of Europe, remains difficult to gauge.

Key research gaps are: (i) assessment of the impact of climate change on diseases that are not vector-borne, including respiratory infections; (ii) evaluation of the vector potential of UK native species; (iii) development of climate driven models for the risk of West Nile and Lyme in the UK; (iv) evaluation of the threat posed by TBE to the UK and (v) incorporation of environmental and agricultural variables into models of the risk of vivax malaria in the UK.

Competing interests

The author declares there are no competing interests

DIsclaimer

The views expressed are those of the author(s) and not necessarily those of the NHS, the NIHR, the Department of Health or Public Health England.

References

1. Morens DM, Folkers GK, Fauci AS: The challenge of emerging and re-emerging infectious diseases. *Nature* 2004, 430(6996):242-249.
2. McIntyre KM, Setzkorn C, Hepworth PJ, Morand S, Caminade C, Morse AP *et al*: Risk assessment of climate-change impacts on infectious diseases in Europe. *Nature Climate Change* 2016, submitted.
3. Lindgren E, Andersson Y, Suk JE, Sudre B, Semenza JC: Monitoring EU Emerging Infectious Disease Risk Due to Climate Change. *Science* 2012, 336(6080):418-419.
4. Cann K, Thomas DR, Salmon R, Wyn-Jones A, Kay D: Extreme water-related weather events and waterborne disease. *Epidemiol Infect* 2013, 141(04):671-686.
5. Eze J, Scott E, Pollock K, Stidson R, Miller C, Lee D: The association of weather and bathing water quality on the incidence of gastrointestinal illness in the west of Scotland. *Epidemiol Infect* 2014, 142(06):1289-1299.
6. Jones KE, Patel NG, Levy MA, Storeygard A, Balk D, Gittleman JL *et al*: Global trends in emerging infectious diseases. *Nature* 2008, 451(7181):990-993.
7. Kuhn KG, Campbell-Lendrum DH, Armstrong B, Davies CR: Malaria in Britain: Past, present, and future. *Proc Natl Acad Sci U S A* 2003, 100(17):9997-10001.
8. Danis K, Baka A, Lenglet A, Van Bortel W, Terzaki I, Tseroni M *et al*: Autochthonous Plasmodium vivax malaria in Greece, 2011. *Eurosurveillance* 2011, 16(42).
9. EFSA/ECDC: The European Union Summary Report on Trends and Sources of Zoonoses, Zoonotic Agents and Food-borne Outbreaks in 2012. *EFSA Journal* 2014, 12(2):1-312.
10. Morin CW, Comrie AC: Regional and seasonal response of a West Nile virus vector to climate change. *Proc Natl Acad Sci U S A* 2013, 110(39):15620-15625.
11. Pradier S, Lecollinet S, Leblond A: West Nile virus epidemiology and factors triggering change in its distribution in Europe. *Rev Sci Tech OIE* 2012, 31(3):829-844.
12. Medlock JM, Vaux AGC: Distribution of West Nile virus vector, Culex modestus, in England. *Vet Rec* 2012, 171(11).
13. Parker DE, Legg TP, Folland CK: A New Daily Central England Temperature Series, 1772-1991. *Int J Climatol* 1992, 12(4):317-342.
14. Mellor PS, Carpenter S, Harrup L, Baylis M, Mertens PPC: Bluetongue in Europe and the Mediterranean Basin: History of occurrence prior to 2006. *Prev Vet Med* 2008, 87(1-2):4-20.
15. Mackenzie-Impoinvil L, Impoinvil DE, Galbraith SE, Dillon RJ, Ranson H, Johnson N *et al*: Evaluation of a temperate climate mosquito, Ochlerotatus detritus (= Aedes detritus), as a potential vector of Japanese encephalitis virus. *Med Vet Entomol* 2015, 29(1):1-9.
16. Angelini R, Finarelli AC, Angelini P, Po C, Petropulacos K, Silvi G *et al*: Chikungunya in north-eastern Italy: a summing up of the outbreak. *Eurosurveillance* 2007, 12(47).

17. Grandadam M, Caro V, Plumet S, Thiberge J-M, Souares Y, Failloux A-B *et al*: Chikungunya Virus, Southeastern France. *Emerg Infect Dis* 2011, 17(5):910-913.
18. La Ruche G, Souares Y, Armengaud A, Peloux-Petiot F, Delaunay P, Despres P *et al*: First two autochthonous dengue virus infections in metropolitan France, September 2010. *Eurosurveillance* 2010, 15(39):2-6.
19. Gjenero-Margan I, Aleraj B, Krajcar D, Lesnikar V, Klobucar A, Pem-Novosel I *et al*: Autochthonous dengue fever in Croatia, August-September 2010. *Eurosurveillance* 2011, 16(9):2-5.
20. Marchand E PC, Jeannin C, Lafont E, Bergmann T, Flusin O, Rizzi J, Roux N, Busso V, Deniau J, Noel H, Vaillant V, Leparc-Goffart I, Six C, Paty MC: Autochthonous case of dengue in France, October 2013. *Eurosurveillance* 2013, 18(50).
21. Almeida A, Gonçalves Y, Novo M, Sousa C, Melim M, AJ G: Vector monitoring of Aedes aegypti in the Autonomous Region of Madeira, Portugal. *Eurosurveillance* 2007, 12(46).
22. Sousa CA, Clairouin M, Seixas G, Viveiros B, Novo MT, Silva AC *et al*: Ongoing outbreak of dengue type 1 in the Autonomous Region of Madeira, Portugal: preliminary report. *Eurosurveillance* 2012, 17(49):15-18.
23. Chouin-Carneiro T, Vega-Rua A, Vazeille M, Yebakima A, Girod R, Goindin D *et al*: Differential Susceptibilities of Aedes aegypti and Aedes albopictus from the Americas to Zika Virus. *PLoS Negl Trop Dis* 2016, 10(3):e0004543.
24. Foy BD, Kobylinski KC, Chilson Foy JL, Blitvich BJ, Travassos da Rosa A, Haddow AD *et al*: Probable non-vector-borne transmission of Zika virus, Colorado, USA. *Emerg Infect Dis* 2011, 17(5):880-882.
25. Dujardin JC, Campino L, Canavate C, Dedet JP, Gradoni L, Soteriadou K *et al*: Spread of vector-borne diseases and neglect of leishmaniasis, Europe. *Emerg Infect Dis* 2008, 14(7):1013-1018.
26. Ready PD: Leishmaniasis emergence in Europe. *Eurosurveillance* 2010, 15(10):29-39.
27. Shaw SE, Langton DA, Hillman TJ: Canine leishmaniosis in the United Kingdom: A zoonotic disease waiting for a vector? *Vet Parasitol* 2009, 163(4):281-285.
28. Stefanoff P, Parda N, Polkowska A, D'Anconca F, Anda P, Rizzoli A *et al*: Epidemiological situation of tick-borne encephalitis in the European Union and European Free Trade Association countries. In: *ECDC Technical Report*. Stockholm: European Centre for Disease Prevention and Control; 2012.
29. Randolph SE: Evidence that climate change has caused 'emergence' of tick-borne diseases in Europe? *Int J Med Microbiol* 2004, 293:5-15.
30. Sumilo D, Asokliene L, Bormane A, Vasilenko V, Golovljova I, Randolph SE: Climate Change Cannot Explain the Upsurge of Tick-Borne Encephalitis in the Baltics. *PLoS One* 2007, 2(6).
31. Lindgren E, Talleklint L, Polfeldt T: Impact of climatic change on the northern latitude limit and population density of the disease-transmitting European tick Ixodes ricinus. *Environ Health Perspect* 2000, 108(2):119-123.

32. Maltezou HC, Andonova L, Andraghetti R, Bouloy M, Ergonul O, Jongejan F *et al*: Crimean-Congo hemorrhagic fever in Europe: current situation calls for preparedness. *Eurosurveillance* 2010, 15(10):48-51.
33. Jameson LJ, Morgan PJ, Medlock JM, Watola G, Vaux AGC: Importation of Hyalomma marginatum, vector of Crimean-Congo haemorrhagic fever virus, into the United Kingdom by migratory birds. *Ticks Tick Borne Dis* 2012, 3(2):95-99.
34. Atkinson B, Latham J, Chamberlain J, Logue C, O'Donoghue L, Osborne J *et al*: Sequencing and phylogenetic characterisation of a fatal Crimean - Congo haemorrhagic fever case imported into the United Kingdom, October 2012. *Eurosurveillance* 2012, 17(48):7-10.
35. McMichael AJ, Campbell-Lendrum D, Kovats S, Edwards SJ, Wilkinson P, Wilson T *et al*: Global Climate Change. Global and Regional Burden of Disease Attributable to Selected Major Risk Factors. In: *Comparative Quantification of Health Risks: Global and Regional Burden of Disease Attributable to Selected Major Risk Factors.* Edited by Ezzati M, Lopez AD, Rodgers A, Murray CJL, vol. 2. Geneva: World Health Organization; 2004: 1543-1649.
36. Hales S, Kovats S, Lloyd S, Campbell-Lendrum D (eds.): Quantitative risk assessment of the effects of climate change on selected causes of death, 2030s and 2050s. Geneva: World Health Organisation; 2014.
37. Caminade C, Kovats S, Rocklov J, Tompkins AM, Morse AP, Colón-González FJ *et al*: Impact of climate change on global malaria distribution. *Proc Natl Acad Sci U S A* 2014, 111(9):3286-3291.
38. Lindsay SW, Hole DG, Hutchinson RA, Richards SA, Willis SG: Assessing the future threat from vivax malaria in the United Kingdom using two markedly different modelling approaches. *Malar J* 2010, 9:70.
39. Lindsay SW, Thomas CJ: Global warming and risk of vivax malaria in Great Britain. *Global Change and Human Health* 2001, 2(1):80-84.
40. Rogers D, Hay SI: The climatic suitability for dengue transmission in continental Europe. In: *ECDC Technical Report.* Stockholm: European Centre for Disease Prevention and Control; 2012: 1-22.
41. Caminade C, Medlock JM, Ducheyne E, McIntyre KM, Leach S, Baylis M *et al*: Suitability of European climate for the Asian tiger mosquito Aedes albopictus: recent trends and future scenarios. *J R Soc Interface* 2012, 9(75):2708-2717.
42. Fischer D, Thomas SM, Suk JE, Sudre B, Hess A, Tjaden NB *et al*: Climate change effects on Chikungunya transmission in Europe: geospatial analysis of vector's climatic suitability and virus' temperature requirements. *Int J Health Geogr* 2013, 12.
43. Thomas SM, Fischer D, Fleischmann S, Bittner T, Beierkuhnlein C: Risk assessment of dengue virus amplification in europe based on spatio-temporal high resolution climate change projections. *Erdkunde* 2011, 65(2):137-150.
44. Ogden NH, Maarouf A, Barker IK, Bigras-Poulin M, Lindsay LR, Morshed MG *et al*: Climate change and the potential for range expansion of the Lyme disease vector Ixodes scapularis in Canada. *Int J Parasitol* 2006, 36(1):63-70.
45. Gilbert L: Altitudinal patterns of tick and host abundance: a potential role for climate change in regulating tick-borne diseases? *Oecologia* 2010, 162(1):217-225.

46. Rizzoli A, Hauffe HC, Carpi G, Vourc'h GI, Neteler M, Rosa R: Lyme borreliosis in Europe. *Eurosurveillance* 2011, 16(27):2-9.
47. Lindgren E, Jaenson TGT: Lyme borreliosis in Europe: influences of climate and climate change, epidemiology, ecology and adaption measures. In. Geneva: World Health Organization; 2006.
48. Ayres J, Forsberg B, Annesi-Maesano I, Dey R, Ebi K, Helms P *et al*: Climate change and respiratory disease: European Respiratory Society position statement. *Eur Respir J* 2009, 34(2):295-302.

Author response

Dear Dr Ebi,

Potential impact of climate change on emerging infections in the UK, by Matthew Baylis, University of Liverpool.

Thank you for your email of 22 January 2017 enclosing referees' reports on my manuscript and requesting that I provide a revision. I am delighted to attach a track-changed version of the original paper, a new final paper and, below, a response to the comments of the two referees. In particular, I have updated some information to take account of important developments since the paper's submission.

Yours sincerely,
Matthew Baylis

Reviewer 1

Page 2, comment [1]: Reference added

Page 2, requested insertion. Text inserted

Page 2, [2]: Reference added

Page 2 [4]. I have modified the text to make the point that climate (and climate change) may have played a role in terms of creating a suitable environment for transmission after pathogen introduction.

Page 2, [5]. Text amended.

Page 3, [1.2]. Text amended to make a point similar to 2 [4] above, and reference added.

Page 4, [1]. References corrected

Page 4, [2,3]. Text corrected

Page 4, requested insertion. Text inserted

Page 4, [4]. I agree that there are more recent and Europe-focused references but, on examination, it seems that these do not alter the conclusions significantly enough to warrant changing the manuscript. I am hesitant about simply adding references to papers of the referees themselves.

Page 7, [1]. Point taken but, until it becomes officially notifiable, the text should remain as is.

Page 8, [1]. Text amended as requested.

Reviewer 2

Page 1 [1]. I have amended the title to indicate that the primary focus of the paper is vector-borne infections. I have not changed the reference to the UK. Much of the information provided is about Europe but it was selected mostly for its relevance to UK risk. I cannot claim that this paper is a detailed assessment of the risk to Europe – for which I would need to consider the situation in North Africa and western Asia.

Page 4, [1] – no reponse needed

Page 4, [2] – the text has been amended, as per the suggestion of Semenza.

Page 4, [3]. Text amended to now refer to 2017 also

Page 4, [4]. More information has been provided on the risks of Zika transmission by the two species of Aedes, including a new reference. I have also added elsewhere that Ae aegypti has also been detected in southeast Europe (Georgia), and that Ae albopictus has now been detected in the UK for the first time.

Page 4, [5]. Reference added

Page 6, [1]. In my view, this point is made in the previous paragraph – ie, that vivax malaria has been present in northern Europe before. I have clarified this by adding 'northern Europe' rather than just referring to the UK.

Page 6, [2]. Point taken, but I do not think the text needs amending.

Page 6, [3]. I have added two of these references and amended the text to provide more information on dengue models.

Page 7, [1]. I have added a line about forecasting (as a subset of risk assessment)

Page 8, [1]. The paper makes only one reference to climate change impacts to respiratory disease. The conclusion is that this is a research gap, and so I think this is the right place to mention it.
